# Supplementary material for: Safety of perioperative treatment with biologics in patients with inflammatory bowel disease undergoing bowel surgery: Experience from a large urban center
Source: PLoS One. 2024 Jan 18;19(1):e0290887. doi: 10.1371/journal.pone.0290887 (PMC10796016; doi:10.1371/journal.pone.0290887)
Supplement: Attachment — Submitted filename: Response to reviewers version 04082023.doc [file pone.0290887.s003.doc]

| PD Dr. Fabian Schnitzler  Klinikum der Universität München · Med. Klinik und Poliklinik II ·  Marchioninistr. 15 · 81377 München | |  |  |
| --- | --- | --- | --- |
| **To the**  **Managing Editors of PLOS ONE** | |  |  |
|  |  |  | August 4^th^ 2023 |

Dear Professor Clementi,

Thank you for consideration of our Manuscript PONE-D-23-13172 “Safety of perioperative treatment with biologics in patients with inflammatory bowel disease undergoing bowel surgery: experience from a large urban center”.

Please find attached below the point-by-point answers to the comments of the reviewer of our manuscript, as well as the manuscript with corrections and a clean copy version.

We hope that our actual manuscript with the corrections now fulfills the requirements of Plos One.

Thank you for re-consideration of our corrected manuscript for publication to Plos One.

With kindest regards,

Fabian Schnitzler

1. Please ensure that your manuscript meets PLOS ONE's style requirements, including those for file naming. The PLOS ONE style templates can be found at 
<https://journals.plos.org/plosone/s/file?id=wjVg/PLOSOne_formatting_sample_main_body.pdf> and 
<https://journals.plos.org/plosone/s/file?id=ba62/PLOSOne_formatting_sample_title_authors_affiliations.pdf>

**We thank the Editors for this important comment. The citation style was now adapted to the PLOS ONE style templates.**

2. Thank you for stating the following financial disclosure: 
"This work was financially supported by the European Crohn’s and Colitis Foundation
(ECCS) and HEXAL AG"

Please state what role the funders took in the study.  If the funders had no role, please state: "The funders had no role in study design, data collection and analysis, decision to publish, or preparation of the manuscript." 
If this statement is not correct you must amend it as needed. 
Please include this amended Role of Funder statement in your cover letter; we will change the online submission form on your behalf.

**We thank the Editors for this comment. The funders had no role in our study. Hence, now we included the statement “The funders had no role in study design, data collection and analysis, decision to publish, or preparation of the manuscript.” in the paragraph “Funding” of our manuscript.**

3. Thank you for stating the following in your Competing Interests section:  
"NO authors have competing interests"

Please complete your Competing Interests on the online submission form to state any Competing Interests. If you have no competing interests, please state "The authors have declared that no competing interests exist.", as detailed online in our guide for authors at <http://journals.plos.org/plosone/s/submit-now> 
 This information should be included in your cover letter; we will change the online submission form on your behalf.

**We completed the Competing Interests of the authors now on the online submission form for all authors as follows:**

- T**. Ochsenkühn has received travel, research and study grants or honoraria for consultations or lectures from AbbVie, Amgen, Biogen, Celltrion, Fresenius, Hexal, Janssen, Lilly, MSD, Viatris, Vifor, Pfizer and Takeda.**
- **F. Schnitzler has received travel grants and honoraria for consultations or lectures from Abbvie, Amgen, Biogen, Hexal, Janssen, Lilly, MSD, Viatris, Pfizer and Takeda.**
- **C. Tillack has received travel grants or honoraria for consultations from Janssen, Galapagos and Lilly.**
- **D. Szokodi has received travel grants or honoraria for consultations or lectures from AbbVie, Amgen, Biogen, BMS, Celltrion, Shire, Lilly, Janssen, Pfizer and Takeda.**
- **M. Sohn has received honoraria for lectures from Galapagos, Lilly, and Taked.**
- **F. Bader has received travel, research and study grants or honoraria for consultations or lectures from Intuitive Surgical Medtronic, MTIGER and Takeda**
- **C. Waggershauser has received travel grands and lecture fees from Biogen, Janssen and Lilly.**
- **This did not alter the authors' adherence to all the PLOS ONE policies on sharing data and materials, as detailed online in the guide for authors.**
- **The other authors have no conflicts of interest to disclose.**
- **No writing assistance was utilized in the production of this manuscript.**
- **There are no patents, products in development or marketed products to declare.**

4. We note that you have indicated that data from this study are available upon request. PLOS only allows data to be available upon request if there are legal or ethical restrictions on sharing data publicly. For more information on unacceptable data access restrictions, please see [http://journals.](http://journals.plos.org/plosone/s/data-availability#loc-unacceptable-data-access-restrictions)plos.org/plosone/s/data-availability#loc-unacceptable-data-access-restrictions.

In your revised cover letter, please address the following prompts:

a) If there are ethical or legal restrictions on sharing a de-identified data set, please explain them in detail (e.g., data contain potentially sensitive information, data are owned by a third-party organization, etc.) and who has imposed them (e.g., an ethics committee). Please also provide contact information for a data access committee, ethics committee, or other institutional body to which data requests may be sent.

b) If there are no restrictions, please upload the minimal anonymized data set necessary to replicate your study findings as either Supporting Information files or to a stable, public repository and provide us with the relevant URLs, DOIs, or accession numbers. For a list of acceptable repositories, please see [http://journals.](http://journals.plos.org/plosone/s/data-availability#loc-recommended-repositories)plos.org/plosone/s/data-availability#loc-recommended-repositories.

We will update your Data Availability statement on your behalf to reflect the information you provide.

**Thank you for this important comment. We again discussed this point with the statisticians. There was a misunderstanding. All relevant data for the paper are already included in the tables of the manuscript. Even more, all data are included for future meta-analyses. We updated this information in the submission site: “*Yes, all data are fully available without restrictions.”* And *“All relevant data are within the manuscript and its Supporting Information files.”* Now we uploaded the data of the manuscript as supporting information files.**

5. Please review your reference list to ensure that it is complete and correct. If you have cited papers that have been retracted, please include the rationale for doing so in the manuscript text, or remove these references and replace them with relevant current references. Any changes to the reference list should be mentioned in the rebuttal letter that accompanies your revised manuscript. If you need to cite a retracted article, indicate the article’s retracted status in the References list and also include a citation and full reference for the retraction notice.

**We again reviewed our reference list. It is complete and correct. Thank you for this comment.**

Additional Editor Comments:

Minor revisions are requested


[Note: HTML markup is below. Please do not edit.]

Reviewers' comments:

Reviewer's Responses to Questions

**Comments to the Author**

1. Is the manuscript technically sound, and do the data support the conclusions?

The manuscript must describe a technically sound piece of scientific research with data that supports the conclusions. Experiments must have been conducted rigorously, with appropriate controls, replication, and sample sizes. The conclusions must be drawn appropriately based on the data presented.

Reviewer #1: Yes

Reviewer #2: Yes

2. Has the statistical analysis been performed appropriately and rigorously?

Reviewer #1: Yes

Reviewer #2: Yes

3. Have the authors made all data underlying the findings in their manuscript fully available?

The [PLOS](http://www.plosone.org/static/policies.action#sharing) Data policy requires authors to make all data underlying the findings described in their manuscript fully available without restriction, with rare exception (please refer to the Data Availability Statement in the manuscript PDF file). The data should be provided as part of the manuscript or its supporting information, or deposited to a public repository. For example, in addition to summary statistics, the data points behind means, medians and variance measures should be available. If there are restrictions on publicly sharing data—e.g. participant privacy or use of data from a third party—those must be specified.

Reviewer #1: Yes

Reviewer #2: Yes

4. Is the manuscript presented in an intelligible fashion and written in standard English?

PLOS ONE does not copyedit accepted manuscripts, so the language in submitted articles must be clear, correct, and unambiguous. Any typographical or grammatical errors should be corrected at revision, so please note any specific errors here.

Reviewer #1: Yes

Reviewer #2: No

5. Review Comments to the Author

Please use the space provided to explain your answers to the questions above. You may also include additional comments for the author, including concerns about dual publication, research ethics, or publication ethics. (Please upload your review as an attachment if it exceeds 20,000 characters)

Reviewer #1: Many thanks to the authors for giving me the opportunity to revise this article. This is a retrospective analysis about the treatment with biologics before surgery in IBD patients.

Some comments:

- why postoperative complications were not classify according to Clavien-Dindo classification?

**Thank you for this important comment. We did not classify the postoperative outcomes according to the Clavien-Dindo classification according to its retrospective study design. We discussed this point with our surgeons. We could not identify hundred percent of information needed to fulfill the Clavien-Dindo classification. Hence, the classification would not be as precise as needed. Based on the large prospective PUCCINI trial (Cohen BL et al. Gastroenterology 2022; 163:204–221) we choose a descriptive classification best depicting our available data from the patients’ charts.**

Reviewer #2: dear Authors, thanks for the opportunity to review your work. I congratulate with you all for the well conducted and interesting investigation. Here below my comments:
Intestinal paralysis meant prolonged postoperative ileus? for how many days before flatus or oral food intake? please clarify

**Intestinal paralysis means a prolonged postoperative ileus and is defined as a dysmotility of the small bowel and the colon for more than 72 hours in combination with nausea, and/or vomiting, abdominal pain, and abdominal distension. This is now clarified in the actual version of the paper in the paragraph “End points and statistical analysis” on pages 7 and 8 *:” Intestinal paralysis was seen as a prolonged postoperative ileus and is defined as a dysmotility of the small bowel and the colon for more than 72 hours in combination with nausea, and/or vomiting, abdominal pain, and abdominal distension.”***

Nausea meant a symptom associated with the above? or do you mean incidence of PONV?

**Nausea meant a symptom associated with the above and not PONV.**

Insufficiency means anastomotic leak? subclinical or overt disruption? please give details and clarify

**Anastomotic insufficiency meant anastomotic leackage. Postoperative intraabdominell abscess, fistula, peritonitis, and anastomotic leackage are subsumed as postoperative intrabdominal septic complications (IASC).**

**Thereof, we selected anastomotic leackage for more detailed information on postoperative complications, which is** **defined as overt disruption of the stapler or suture line, identified by endoscopic or surgical exploration.**

**This is now clearly mentioned in the actucal version of the paper, in the paragraph “End points and statistical analysis” on page 8: “*Anastomotic insufficiency was seen as anastomotic leakage and defined as overt disruption of the stapler or suture line, identified by endoscopic or surgical exploration*”.**

CD patients showed almost threefold increase in anastomotic leak rate compared to UC (17 vs 6). was it statistically significant? please make a comment on this.

**In a total of 17 of 334 CD patients occurred anastomotic leakage (5.09%) compared to a total of 6 UC patients in the UC cohort of 113 patients (5.31%). This was not statistically significant (p= 1.000, chi square test). This is now mentioned in the paper on page 15, in the paragraph “Major complications”: No significant difference was observed in CD patients versus UC patients regarding anastomotic insufficiency (CD, 17/334 patients, 5.09%, vs. UC, 6/113 patients, 5.31%, p=1.000, table 2).**

Some sentences are a bit complicate and confusing. please make a full evaluation by a mother language.

**We asked a native English speaker to read the final version of our manuscript and to make corrections. Thank you for the comment.**

6. PLOS authors have the option to publish the peer review history of their article ([what does this mean?](https://journals.plos.org/plosone/s/editorial-and-peer-review-process#loc-peer-review-history)). If published, this will include your full peer review and any attached files.


If you choose “no”, your identity will remain anonymous but your review may still be made public.


**Do you want your identity to be public for this peer review?** For information about this choice, including consent withdrawal, please see our [Privacy Policy](https://www.plos.org/privacy-policy).

Reviewer #1: No

Reviewer #2: No

[NOTE: If reviewer comments were submitted as an attachment file, they will be attached to this email and accessible via the submission site. Please log into your account, locate the manuscript record, and check for the action link "View Attachments". If this link does not appear, there are no attachment files.]

While revising your submission, please upload your figure files to the Preflight Analysis and Conversion Engine (PACE) digital diagnostic tool, <https://pacev2.apexcovantage.com/>. PACE helps ensure that figures meet PLOS requirements. To use PACE, you must first register as a user. Registration is free. Then, login and navigate to the UPLOAD tab, where you will find detailed instructions on how to use the tool. If you encounter any issues or have any questions when using PACE, please email PLOS at [figures@plos.org](mailto:figures@plos.org). Please note that Supporting Information files do not need this step.
